# Investigating Challenges in Implementing a Digital Play Intervention in a Complex Organization Across Pediatric Departments: Non-Randomized Controlled Feasibility Trial

**DOI:** 10.2196/58019

**Published:** 2025-07-08

**Authors:** Laerke Winther, Michelle Stahlhut, Derek John Curtis, Christian Have Dall, Thomas Leth Frandsen, Jette Led Sorensen

**Affiliations:** 1 Mary Elizabeth’s Hospital - Rigshospitalet for Children, Teens and Expecting Families, Juliane Marie Centre, Copenhagen University Hospital, Rigshospitalet, Blegdamsvej 9, Copenhagen, 2100, Denmark, 45 35458901; 2 Centre for Clinical Research and Prevention, Copenhagen University Hospital, Bispebjerg and Frederiksberg Hospital, Copenhagen, Denmark; 3 Child Centre Copenhagen, The Child and Youth Administration, Copenhagen, Denmark; 4 Department of Occupational Therapy and Physiotherapy, Copenhagen University Hospital, Bispebjerg and Frederiksberg Hospital, Copenhagen, Denmark; 5 Department of Clinical Medicine, University of Copenhagen, Copenhagen, Denmark

**Keywords:** hospital, pediatric, toddler, child, youth, adolescent, teenager, physical activity, feasibility, mHealth, mobile health, mobile app, digital health, digital technology, digital intervention, digital play, smartphone, exergame, digital game

## Abstract

**Background:**

Patients and health care providers can use playful digital games in a hospital setting to increase motivation and distract patients during painful procedures. Future digital interventions for pediatric hospitalization must do more than distract; they must also encourage socialization and promote physical activity, for example, by exploring novel interactive approaches to boost motivation.

**Objective:**

The pilot study investigated the feasibility of a non-randomized controlled trial (non-RCT) assessing a new digital play intervention, Monster Gardener, that aims to motivate and increase physical activity for children and adolescents in a hospital.

**Methods:**

This feasibility study was a non-RCT conducted from October to December 2023. We recruited hospitalized children, 7‐17 years of age, and health care professionals from 4 pediatric departments at Copenhagen University Hospital – Rigshospitalet, Denmark. The children were allocated to intervention and control groups. Data collection included physical activity data measured with accelerometers, data on app use, and usability questionnaires completed by participants and health care professionals. The control group received usual care and accelerometer measurements, while the intervention group received accelerometer measurements and was invited to play Monster Gardener. We applied the 8 focus areas by Bowen et al to describe and evaluate the app’s feasibility.

**Results:**

A total of 22 children and adolescents from 3 pediatric departments agreed to participate. Our main findings, based on the framework by Bowen et al, were (1) acceptability: prolonged recruitment due to fewer hospital stays more than 24 hours than expected; (2) demand: software coding error in the app prevented data registration, causing a potentially major risk of data loss; (3) practicality: Monster Gardener was incompatible with certain mobile phones, and discomfort from the adhesive plasters used to attach the accelerometer led to early removal by one-third of participants; (4) implementation: technical problems and perceived complexity hindered successful app deployment; (5) adaptation: the app demonstrated adaptability across different departments; (6) integration: enhanced information sessions with the health care professionals were needed prior to data collection, and participants were too exhausted and overwhelmed by consultations, blood tests, examinations, and pain and nausea from surgical procedures to use the app; (7) expansion: app facilitation requires additional resources, posing a challenge given limited availability of staff; and (8) limited-efficacy testing: participants were inactive 22 hours a day and data loss limited efficacy testing.

**Conclusions:**

The digital play intervention showed that Monster Gardener could potentially motivate children to be physically active during pediatric hospitalization; however, when using the framework by Bowen et al, the current version was deemed infeasible for implementation in an RCT. Various organizational, technological, and practical issues must be addressed to improve the intervention prior to effectiveness testing. Future studies should use simpler digital play interventions and invite end users’ active involvement in developing the intervention.

## Introduction

Emerging technologies can be used to create playful digital interventions for children and adolescents. Patients and health care providers can use digital games in a hospital setting to increase motivation, distract patients during painful procedures, and encourage social interaction [1]. Game technologies can improve children’s psychosocial health [1,2]; however, a lack of knowledge exists regarding the types of games that should be used. A recent review of game technologies for pediatric patients showed that the most common approach is mono-user games on ordinary computers or video consoles, for example, PlayStation and Nintendo Wii, that often serve to distract children undergoing procedures such as venipuncture or who experience chronic, neurological, or traumatic diseases or injuries [1]. The review recommends that future digital interventions for pediatric hospitalization should do more than distract; they must also promote socialization and mobility and exploit new technologies, such as robots and interactive features that encourage intrinsic motivation [1].

During hospitalization, children and adolescents often exhibit excessive sedentary behavior, primarily engaging in low-intensity activities [3]. While studies on adults indicate that immobilization during hospitalization can lead to heightened deconditioning, clinical complications (eg, pneumonia, venous thromboembolism, and constipation), and prolonged recovery [4-7], similar research on children is minimal. Reduced physical activity can lead to reduced functional capacity in musculoskeletal and cardiovascular systems, jeopardizing functional independence, which is a crucial factor for hospital discharge and the foundation for postinjury or illness recovery [4,8,9]. Early mobilization has repeatedly been proven safe and feasible for critically ill children and adults with stable cardiorespiratory function [10-13].

Increasing physical activity in sedentary hospitalized children and adolescents is considered beneficial [14]. Play can facilitate increased physical activity. A recent scoping review on play interventions in hospitals finds that play involving physical activity improves coping with hospitalization, disease, and sequelae [15] and could potentially benefit children’s health during hospitalization. Another scoping review finds that various clinical settings have used digital play in rehabilitation but emphasizes the need to investigate further digital play interventions for early rehabilitation of hospitalized children [16]. Mobile apps combining games with physical activity, such as Pokémon Go, can facilitate substantial short-term increases in physical activity [2,17]. Pokémon Go, however, is not designed to accommodate children with functional limitations, such as casts, IV lines, catheters, and walking aids.

This study investigated the feasibility of a customized digital play intervention via a mobile application for hospitalized children and adolescents using the framework by Bowen et al [18], including these 8 focus areas of acceptability, demand, practicality, implementation, adaptation, integration, expansion, and limited efficacy testing. The study preceded a comprehensive randomized controlled trial (RCT) aimed at exploring the impact of the application.

## Methods

### Design

This small-scale feasibility study is a non-RCT conducted from October to December 2023. As noted, we applied the 8 focus areas by Bowen et al [18] to describe and evaluate the feasibility of a digital play intervention called Monster Gardener. The data from this feasibility study were reported using the CONSORT (Consolidated Standards of Reporting Trials) statement extension to pilot and feasibility trials [19].

### Setting and Subjects

Prior to recruitment, we proactively contacted all chief nurses across pertinent departments to secure their approval before proceeding. We then sought endorsement and permission from departmental head nurses, chief pediatric surgeons, and chief pediatricians. To ensure awareness among the clinical nurses, we personally visited all the departments to provide information about the project. Standardized information was disseminated in departmental newsletters, and informative posters were strategically placed in, for example, staff break rooms and offices. Two health care professionals from each department volunteered to be project ambassadors, fostering a collaborative and informed environment.

Initially, we recruited hospitalized children 7‐12 years of age but expanded the criteria to 7‐17 years of age due to a poor recruitment rate. Convenience sampling was used to recruit participants from the department for children with surgical diseases of the abdomen, intestines, or urinary tract; the department for surgical diseases of the face, bones, and joints; the department for children with heart disease; and the department for neuropediatric diseases and children with liver or kidney diseases in a semi-intensive care unit at Rigshospitalet, which is a national tertiary hospital. Children who were in isolation could also participate if they met the eligibility criteria (Textbox 1), which were defined in collaboration with a highly specialized neuropediatrician. The health care professionals treating the patients assessed the eligibility criterion of cognition around 7‐17 years of age, which was chosen to ensure that participants had the mental capacity to understand the intervention and associated questionnaire. We recruited health care professionals who actively engaged in facilitating the digital play intervention in their departments to examine their perspectives on using the app to encourage patients to be more mobile.

Textbox 1. **Eligibility criteria for the children and adolescent study population.**

Inclusion criteria:

- Expected hospitalization >24 hours.
- Able to speak Danish.
- Cognition around 7‐17 years of age.
- Able to use a mobile phone with one hand.
- Wheelchairs and isolation acceptable.

Exclusion criteria:

- Severe uncorrectable visual impairment.
- Neurological or psychiatric disorders that prevent participation.
- Continuous electroencephalogram monitoring.
- Participation in other physical activity studies.

### Intervention

**Figure 1.**
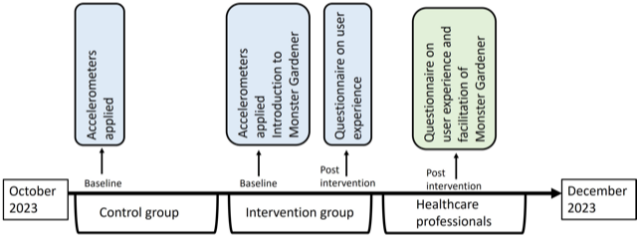
Timeline of the feasibility study. Physical activity in the control group was measured with accelerometers in October 2023. Physical activity in the Monster Gardener group was measured with accelerometers in November 2023. Health care professionals’ user experience was assessed in December 2023.

The first 10 participants were allocated to the control group and the next 10 to the intervention group (Figure 1). Participants in the control group received usual care, which consisted of daily mobilization encouragement and sometimes activities supervised by health care professionals.

The intervention group was invited to play Monster Gardener during their hospitalization. Monster Gardener aimed to motivate and increase physical activity for hospitalized children. Hospitalized children and adolescents, health care professionals, researchers, and external game designers co-designed Monster Gardener in a public-private partnership. The app’s development, which was outsourced to 4 software development and game design companies between 2018 and 2022, underwent continuous testing and evaluation by hospitalized children in an oncology ward, and health care professionals. The companies were responsible for all technical aspects of the app, while the project manager for the app’s design was responsible for clinical testing and providing feedback to the developers.

Augmented reality (AR) technology in the app merges the real world with digital elements, encouraging physical activities like walking and boxing, and the camera lens in users’ phones captures the real world combined with digital elements integrated into the surroundings. Designed to be playful and engaging in countering the negative impacts of hospitalization on children’s health, Monster Gardener invites children to act as gardeners for animated monster plants. The app comprises 6 mini-games: 3 for adaptation (feeding, decorating, and social activities) and 3 five-minute mini-games that encourage physical activity. These games, including 2 walking activities and an activity for the upper extremities, were disguised and embedded in the digital play to encourage movement in an entertaining digital context. AR codes strategically placed in the children’s hospital rooms, around the hospital wards, and on staff uniforms contained food and decorative items for the monster plants that would appear on the children’s phones, nudging them to get out of bed to find the codes. The AR codes, which were color-coded red, yellow, and blue, were positioned in the middle and on either end of the ward. Each quest required scanning at least 2 AR codes to ensure that participants walked from the middle to the end of the ward at a minimum. Upon completing a quest, a new one appeared with an additional AR code to allow participants to advance further. Multimedia Appendix 1 provides additional details. The participants were asked to play the game as frequently as they wished, and the health care professionals and parents were asked to encourage them to play. If a participant did not have a smartphone, they could borrow one from the research team.

### Technology and Data Collection

#### Overview

The children were invited to participate in the study as soon as possible following admission. Data collection continued until discharge or 7 days after inclusion, whichever was the shortest. We aimed for a sample size of at least 10 per arm, referencing Whitehead et al [20].

#### Accelerometers

LW conducted the data collection, which included data on physical activity during hospitalization measured with accelerometers (SENS Motion) embedded in waterproof medical adhesive plasters. Three tri-axial sensors were fitted to each participant’s sternum, lateral aspect of the distal femur, and proximal on the humerus of the dominant arm [21]. The participants were able to engage in usual activities (eg, showering, playing, and sleeping), but the sensors were removed for x-rays, magnetic resonance imaging, and computed tomography scans.

The accelerometers, which continuously sampled accelerations at 12.5 Hz during hospitalization, were connected via Bluetooth to a dedicated app installed on a tablet that uploaded data to a secure server. The device contained onboard memory capacity for approximately 2 weeks to prevent data loss due to occasional lack of connectivity. In the dedicated app, a built-in algorithm detected the accelerometer’s orientation and movements, classifying activities into lying in bed, sitting, standing (including short bouts of shuffling), walking, and the number of steps taken, summarized every 5 seconds. A previous study validating the sensors contains additional technical specifications on the accelerometers [21].

Physical activity was measured in minutes as time in bed (lying down), time spent inactive (sitting or lying down), and time spent out of bed (standing or walking) based on accelerometer wear time, which was reported as the average daily time in or out of bed and being inactive (minutes/day) [22]. Secondary measures included the average daily time spent sitting, standing, and walking, and the average daily number of sit-to-stands and steps per day.

#### Research Electronic Data Capture (REDCap)

To collect data on app use, we used the REDCap (Research Electronic Data Capture; Vanderbilt University) application programming interface (API) feature to automatically import data into the REDCap software [23] using an API key to authenticate requests to ensure security and legitimacy [23]. We also used REDCap to distribute and collect questionnaires.

#### Intrinsic Motivation Inventory (IMI)

When discharged, the participants were asked to complete a questionnaire based on a short version of the Intrinsic Motivation Inventory (IMI) [24] that was translated into Danish using forward-backward translation in a previous study [25] and modified to target children and adolescents. We selected 4 of 7 subscales (interest or enjoyment (motivation), perceived competence, pressure or tension, and perceived choice (autonomy) for our short version, including 12 statements, and modified the scale from 7 to 4 points to make it easier for the youngest participants to respond. To score the IMI, we modified the score for the reversed items by subtracting the response from 5, averaging all item responses on each subscale, where 1=very true and 4=not at all true.

#### Technology Acceptance Model (TAM)

After the last participant in the intervention group was discharged, health care professionals were asked to complete a web-based questionnaire distributed via an iPad on their experience with implementing the intervention in their departments. The questionnaire was based on the Technology Acceptance Model (TAM) [26] and was previously translated into Danish [27]. TAM includes 4 subscales: perceived usefulness, perceived ease of use, attitude, and behavioral intention. We added more study-specific questions on Monster Gardener’s influence on workflow and the use of the accelerometers. All TAM items and additional questions used a 5-point Likert scale, with scores ranging from 1 (strongly agree) to 5 (strongly disagree).

#### Feasibility

To assess the challenges involved in implementing an early-stage digital play intervention, we assessed feasibility using the framework by Bowen et al [18] , including 8 focus areas and used the Consolidated Framework for Implementation Research (CFIR) to analyze implementation potential [28]. Table 1 shows how we organized data collection and outcome measures to investigate the feasibility of the Monster Gardener intervention.

**Table 1.** The 8 focus areas by Bowen et al [18], feasibility considerations, data collection, and data analysis methods.

Eight focus areas and feasibility considerations	Population	Data collection methods	Time of evaluation
Acceptability			
Participant acceptance of the new technology	Children	IMI^a^ (motivation)	Post intervention
Participant acceptance rate to participate	Children	Percentage acceptance	Enrollment
Management’s support, health care professionals’ acceptance of the new technology	Health care professionals	TAM^b^ (attitude)	Post intervention
Demand			
Interest in the app	Children	IMI (autonomy)	Post intervention
Use of the app	Children	Monster Gardener data	Post intervention
Interest in the app	Health care professionals	TAM (behavioral intention)	Post intervention
Implementation			
Degree of likelihood of implementation	Children	CFIR^c^	Post intervention
Degree of likelihood of implementation	Health care professionals	CFIR	Post intervention
Practicality			
Technological aspects of the app	N/A^d^	Fieldnotes, observation	Post intervention
Technological aspects of data collection	N/A	Fieldnotes, observation	Post intervention
Accelerometers	N/A	Fieldnotes, observation	Post intervention
Adaptation			
Degree of similar outcomes in a new format	Children	SENS^e^ activity data	Post intervention
Degree of similar outcomes in a new format	Health care professionals	Study-specific questionnaire (workflow modifications)	Post intervention
Integration			
Perceived fit with infrastructure	Children	Fieldnotes, observation	Post intervention
Perceived fit with infrastructure	Health care professionals	Study-specific questionnaire, fieldnotes, observation	Post intervention
Expansion			
Fit with organizational goals and culture	Children	SENS and use	Post intervention
Fit with organizational goals and culture	Health care professionals	Study-specific questionnaire	Post intervention
Limited efficacy testing^f^			
Intended effect on key intermediate variables	Children	SENS activity data	Post intervention

a IMI: Intrinsic Motivation Inventory.

b TAM: Technology Acceptance Model.

c CFIR: Consolidated Framework for Implementation Research.

d N/A: not applicable.

e SENS: SENS Motion.

f Not applicable to health care professionals.

### Ethical Considerations

This study conforms to the Declaration of Helsinki, was approved by the Danish Data Protection Agency (P-2020‐121), and did not require ethical approval from the National Committee on Health Ethics Research (FSP 21054778). Participants’ parents received an introductory letter accompanied by an information sheet and a consent form. The children provided verbal assent prior to inclusion in the study, and parents provided written informed consent [29]. All data were pseudoanonymized by removing identifiable information and assigning each record a code. The key linking the codes to individual identities was stored securely and separately, in accordance with data protection guidelines.

### Data Analysis

Data analysis was performed using SPSS Statistics (version 29; IBM). All nonnormally distributed data were reported with medians and ranges, and intergroup differences were tested with the Mann-Whitney *U*-Test. Determining reliability was not considered meaningful due to the small sample sizes in IMI and TAM [30].

## Results

### Overview

**Figure 2.**
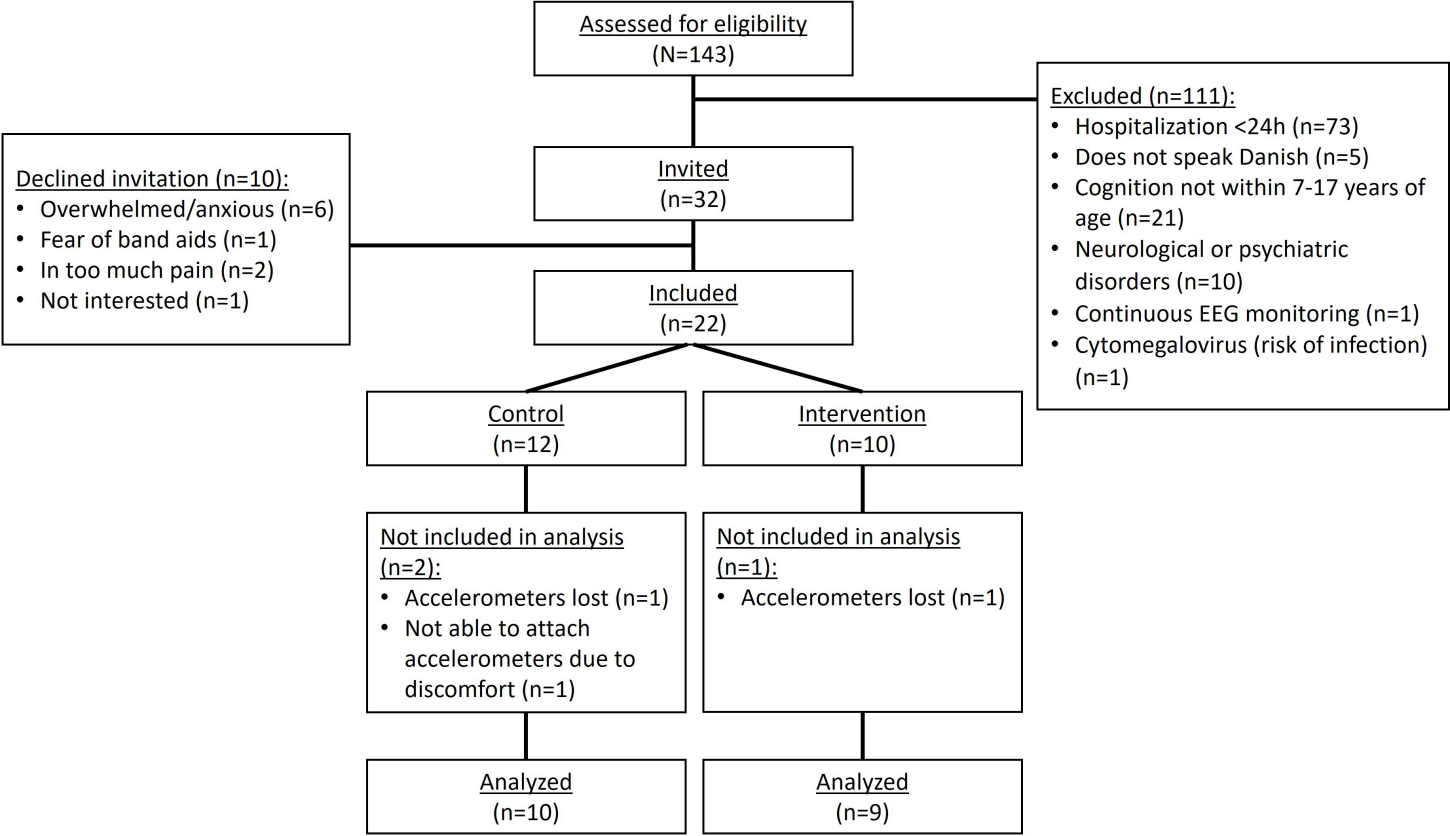
Flowchart of participants in the feasibility study. EEG: electroencephalogram.

There were 143 children from 7 to 17 years of age hospitalized in the 4 pediatric departments from October to December 2023. We excluded 111 patients (Figure 2) and invited 32 to participate, 22 of whom agreed.

The number of participants was 22, with 12 participants in the control group and 10 participants in the intervention group. The median age range was 12 years (7-17 years) for the total, 11 years (7-17 years) for the control group, and 13 years (10-17 years) for the intervention group. Females represented 59% (13 individuals) of the total, of which 67% (8 individuals) were in the control group and 50% (5 individuals) were in the intervention group. Regarding specific medical conditions, 18 participants had surgical diseases affecting bones, joints, or facial structures, with 9 each in the control and intervention groups. Additionally, 3 participants had heart diseases, comprising 2 in the control group and 1 in the intervention group. Last, 1 participant required semi-intensive care, present solely in the control group.

### Acceptability

#### Management’s Support

A total of 10 out of 10 managers (chief pediatric surgeons, chief pediatricians, head pediatric nurses, and head physiotherapists of all departments) approved and supported the project.

#### Acceptance to Participate

We recruited participants from October 3, 2023, to December 8, 2023. Three children per week met the inclusion criteria, but we included 2 a week, yielding a moderate to high participation rate (22/32, 69%). It was difficult to recruit preoperatively as the children were anxious about their imminent medical procedures. Postoperative recruitment was also a challenge due to pain, nausea, and fatigue but it was better tolerated than preoperative recruitment.

#### Participant Acceptance of the New Technology

The children’s motivation to play Monster Gardener and the perceived competence and autonomy while playing were neither positive nor negative, and the children did not feel pressure or anxiety while playing (Table 2).

**Table 2.** Results from the Intrinsic Motivation Inventory (IMI) and the Technology Acceptance Model (TAM).

Questionnaire and subscale	Value, median (range)
IMI (4-point Likert scale), subscale scores (children; n=9)	
Interest or enjoyment (motivation)	2 (1-3)
Perceived competence	2 (1-3)
Tension	4 (3-4)
Perceived choice (autonomy)	2 (1-3)
TAM (5-point Likert scale), subscale scores (health care professionals; n=5)	
Perceived usefulness	1 (1-1)
Perceived ease of use	2 (1-5)
Attitude	2 (1-2)
Behavioral intention	2 (1-2)

#### Health Care Professionals’ Acceptance of the New Technology

When asked about the perceived usefulness, attitude, behavioral intention and perceived ease of use of Monster Gardener, the health care professionals were overall positive (Table 2).

### Demand

#### Use of the App

We integrated the automatic transfer of usage data into REDCap, but it did not function as anticipated. The user account with the API token was unintentionally suspended due to inactivity within REDCap’s given timeframe; therefore, usage data were not registered. An error log to detect this event had mistakenly not been integrated into the software coding. Most of the children told the investigator that they did not play the game very much. Some found it difficult to understand or lost interest due to lagging but most were too exhausted to engage. One family felt it was too soon after general anesthesia. Only 1 adolescent played it enthusiastically while hospitalized.

#### Interest in the App

A total of 7 in the control group and 3 in the intervention group declined to participate, the latter indicating that an appealing intervention creates greater interest in participating. Two from the intervention group who declined to participate reported too much discomfort, and one (girl, 16 y) said it did not appeal to her. The remaining participants found it interesting to participate and were curious about the app.

### Implementation

Authors LW, JLS, DJC, and MS discussed the data and identified the facilitators and barriers shown in Table 3 to implementation based on CFIR [28].

**Table 3.** Barriers and facilitators to implementation based on Consolidated Framework for Implementation Research (CFIR) [28].

	Facilitators to implementation	Barriers to implementation
Innovation (Monster Gardener)	Co-designed with hospitalized children Applied in various clinical settings	Complex, 6 different mini-games and augmented-reality code scan to open new mini-game Incompatible with Android and Motorola phones
Outer setting	Outsourcing to professional game designers and software developers ensured quality of Monster Gardener app	Absence of overall project plan causes lack of overview on competing projects Unintentional outsourcing of operational app data use to software developers
Inner setting	Did not require a specific room or take up space	Wall stickers may not be clearly visible
Individuals	Participants welcomed distraction from waiting time Supportive management and clinicians who emphasized the need for mobilization efforts	Participants too exhausted to play or lacked cognitive capacity to comprehend the app Lack of resources (time, staff shortage) hinders app facilitation
Implementation process	The app’s design allows for easy adaptation across departments due to uniform layouts	Variations in hospitalization characteristics probably make the app most suitable for patients, who have longer stays and are less anxious than first-time patients

### Practicality

#### Technological Aspects of the App

Monster Gardener supports a wide range of mobile phones, but not all (eg, 2 types of Motorola phones were incompatible) and was available in iOS and Android; however, the Android operating system does not support the games with upper extremity activities, thereby hindering the intended levels of physical activity in the app for Android users.

#### Technological Aspects of Data Collection

We used REDCap to gauge participants’ app use and assess the perspectives of participants and health care professionals. Unfortunately, REDCap malfunctioned before data collection commenced, delaying the initiation of the intervention by a week also failed to record actual app use despite direct integration.

We used iPads to collect data on perceived feasibility from participants and health care professionals. If the children were discharged (n=2) or the nurses were too busy (n=1) the questionnaires were distributed by e-mail. The overall response rate was 90% for participants (n=9) and 83% for health care professionals (n=5).

#### Accelerometers

Data were unavailable for 3 participants and limited to 7. Two accelerometer sets were lost—one accidentally taken home and another mistakenly discarded. The third set of missing data was attributed to a participant declining to wear the accelerometers due to discomfort.

A total of 7 of 22 participants removed accelerometers early due to discomfort from the Conformité Europèenne–approved adhesive plasters, for example, itchiness and overall discomfort. One participant, burdened by multiple medical devices, felt overwhelmed, while another declined because they were afraid of the adhesive plasters.

Accelerometers were also removed for computed tomography or magnetic resonance imaging scans, and due to time constraints, reattaching them was not possible, shortening the data collection period. Children who had heart surgery wore the accelerometer on their backs due to surgical wounds on the sternum, resulting in data misclassification with time spent lying down classified in the software as time spent sitting up.

### Adaptation

The app could be adapted to accommodate different needs and situations, although no one in the intervention group was in isolation.

### Integration: Perceived Fit With Infrastructure

Pain, nausea, examinations, and ward rounds left 90% of the children too exhausted to use the app. The study-specific questionnaire based on a 5-point Likert scale asked health care professionals whether the intervention disturbed their usual workflow, with a mean score of 3.2 (SD 0.8) indicating that they neither agreed nor disagreed. When asked if the intervention was unnecessarily time-consuming, they neither agreed nor disagreed (mean 2.6, SD 0.6). They felt that the children were more active when using the app (mean 1.2, SD 0.5).

Several nurses said they had not been told about the study despite our systematic efforts. A lack of a project coordinator across departments posed challenges concerning competing projects. For example, the investigator accidentally discovered an ongoing competing project at the department during data collection for the control group. How many children participated in the competing project or whether it influenced the data in the control group is unknown as it was not a research project, and no data were collected.

### Expansion: Perceived Fit With Organizational Structure

The app’s design makes it appropriate for different departments and hospitals but it requires instruction and facilitation. These resources were limited due to departmental staff shortages that affected all included departments. Management and clinical staff from all departments supported the project; however, the clinical staff did not have the time or capacity to engage further, for example, fix adhesive plasters or facilitate the game. We included no children from one department since it treated primarily infants and toddlers (<4 y).

### Limited Efficacy Testing

The accelerometer data for 19 participants showed that they were inactive (sitting or lying down) for 22 hours a day (lying down 17 h per day). On average the cohort walked 2339 steps daily (Table 4). Between-group differences in accelerometer wear time and sit to stands were statistically significant, but there were no significant between-group differences for other measures.

Table 5 summarizes the main findings for the 8 feasibility areas on levels of concern. No concerns indicate that there is no need for adjustments to the feasibility area; some concerns indicate the need for some adjustments to the area prior to efficacy testing; and serious concerns indicate a strong need for changes.

**Table 4.** Limited efficacy testing of device-based physical activity during hospitalization.

	Total (n=19)	Control group (n=10)	Intervention group (n=9)	*P* value^a^
Hospitalization duration (days), median (range)	4 (1-9)	4 (1-7)	3 (1-9)	.81
Time between hospitalization and recruitment (days), median (range)	1 (0‐6)	1 (0‐5)	2 (0‐6)	.40
Accelerometer wear time (hours), median (range)	42 (2‐126)	62 (26‐126)	26 (2‐48)	.005
Time in bed (minutes per day), median (range)	1082 (68‐1394)	1119 (68‐1366)	961 (455‐1394)	.17
Time inactive (minutes per day), median (range)	1368 (1059‐1423)	1369 (1293‐1423)	1353 (1059‐1415)	.22
Time out of bed (minutes per day), median (range)	58 (1‐141)	52 (1‐134)	58 (5‐141)	.51
Sitting time (minutes per day), median (range)	289 (5‐1233)	264 (5‐1233)	336 (21‐898)	.19
Standing time (minutes per day), median (range)	17 (0‐58)	16 (0‐48)	17 (0‐58)	.84
Walking time (minutes per day), median (range)	33 (0‐119)	21 (0‐119)	41 (5‐101)	.37
Sit to stand (number of times), median (range)	26 (8‐160)	19 (8‐160)	48 (17‐94)	.04
Steps (number), median (range)	1635 (0‐9017)	1062 (10‐9017)	2103 (0‐5443)	.33

a Mann-Whitney *U* test significance. Statistical significance set at <.05.

**Table 5.** Levels of concern regarding the intervention’s feasibility.

Levels of concern in the 8 focus areas by Bowen et al [18]	Degree of concern	Main findings	Proposed actions required before conducting a randomized controlled trial
Acceptability	Some concerns	Prolonged recruitment due to fewer hospitalizations >24h	Use a sample size power calculation from similar studies to determine required number of participants for statistical significance Consider multi-hospital recruitment to meet recommended number of participants
Demand	Serious concerns	Significant risk of data loss due to a lack of awareness of REDCap API regulations and lack of software coding of error log Children too exhausted to play due to overwhelming medical routines	Mitigate risk of operational data loss at hospital or regional level by implementing measures such as dedicated REDCap software service accounts Simplify game intervention and enhance motivational aspects, eg incorporating rewards
Practicality	Serious concerns	Monster Gardener was incompatible with some Motorola phones, 50% of app unavailable on Android phones One-third of the children removed accelerometer adhesive plasters early due to discomfort	Develop Monster Gardener further to address challenges or provide iPhones to test the app further (both costly measures) Explore and test alternative solutions to adhesive plasters or attachment methods like Velcro bands
Implementation	Some concerns	Technical glitches and perceived complexity hindered successful app deployment	Improve internet connection by encouraging participants to use free hospital guest WiFi Introduce app during presurgery conversations for home exploration the day before admission Simplify the app by having fewer features (incurs additional costs)
Adaptation	No concerns	App demonstrated adaptability across different departments	—^a^
Integration	Some concerns	Enhanced information sessions with the health care professionals needed prior to data collection	Conduct information meetings on different days to ensure that all shifts are covered Appoint ambassadors to disseminate project details among colleagues
Expansion	Some concerns	App facilitation requires additional resources, posing a challenge given limited availability of health care professionals	A possible action could be to hire a student or research assistant to facilitate use of the app
Limited efficacy testing	Some concerns	Children inactive for 22 hours a day; however, data loss and a small sample size limited the efficacy testing	Examine alternative ways to attach accelerometers even though the accelerometer technology performed well

a Not applicable.

## Discussion

### Principal Findings

We assessed the feasibility of implementing the customized Monster Gardener app in pediatric departments in a highly specialized tertiary hospital in Denmark. We found that the implementation of the app was not feasible without substantial amendments and identified several areas of concern when implementing digital interventions in a complex organization.

According to the World Health Organization’s guide for monitoring and evaluating digital interventions [31] identifying a digital intervention’s stage of maturity is a critical first step in defining the appropriate evaluation approach. On a continuum from preprototype, prototype, and pilot (early stage) to scaling up (middle) and eventually integrated implementation (advanced), we estimated that Monster Gardener was in the early stage. World Health Organization maintains that the appropriate evaluation approach for interventions in early-stage maturity is testing feasibility, usability, and efficacy. Our feasibility study findings indicate that we correctly estimated the maturity stage and evaluation approach, avoiding premature assessment.

Underreporting of negative results from feasibility studies is estimated to be high [32]. Failure to publish negative results causes publication bias and is unethical, leading to unnecessarily investing resources in interventions already proven to be infeasible [33]. As a result, publishing our negative findings is imperative so that others can learn from them [34].

Overall, this study showed that the acceptability of Monster Gardener differed between management, health care professionals, and hospitalized children. Acceptability was high among management and health care professionals, but the children did not find the app motivating, nor did playing it give them a sense of competence and autonomy, which can contribute to the intrinsic motivation to play [35]. The app’s design or hospitalization may have deterred this motivation as indicated by some children’s statements that it was too complex, or they were too exhausted to play. The recruitment rate also indicated that hospitalization may be overwhelming. In the case of pediatric burn rehabilitation, one study [36] found that a virtual reality intervention was more fun during pediatric burn rehabilitation than standard therapy, while another [37] found no difference between video games and standard therapy. An intervention’s digital nature alone does not guarantee that it will have a motivational impact [16], and the Monster Gardener app is possibly an example of an unmotivating design in this sample of children.

A key finding of this study was identifying the major risk of data loss when using REDCap API to track usage data directly from the app. Using REDCap API for data import from other technologies has great potential for automating data import and controlling data input [38]; however, we were not aware that inactivity in the account holding the API key would lead to suspension of the key. We were unable to identify other researchers with a similar experience, but we believe that awareness of this issue when using the REDCap API feature is particularly relevant.

A similar study on the feasibility of video games to enhance physical activity in children in a pediatric critical care unit found that the video games were not feasible as the children needed to be cognitively alert and able to comply with the intervention [39]. Correspondingly, we found that the children were too exhausted and did not have the capacity to interact with the app.

A recent scoping review finds that studies on digital play and rehabilitation rarely examine implementation options [16]. Using CFIR, we analyzed the barriers and facilitators for implementing our intervention since we aimed to conduct a subsequent RCT. Implementing interventions in complex organizations poses multifaceted challenges [40], and one barrier to implementation was that the nurses were too busy to facilitate the intervention. Another feasibility study comparing using videogames for exercise to standard physical therapy in a pediatric burn unit emphasized that having physical or occupational therapists facilitate the exercises in the videogames ensured safe and appropriate use of the technology with maximum benefit [37]. A recent scoping review on digital play and rehabilitation indicated that interventions that do not occupy physical space facilitate the intervention [16]. During our intervention, the wall stickers with AR codes were occasionally covered by beds, hindering implementation. To address these challenges, intervention development should be considered a dynamic iterative process to be executed in close collaboration with the health care professionals who will ultimately use the intervention, while simultaneously drawing on existing evidence, undertaking primary data collection to understand the context and thoroughly analyzing facilitators and barriers for future implementation [40].

Another significant finding was that certain app features were not compatible with Android phones, causing them to operate differently than expected. Hence, researchers and health care professionals must be aware that the software’s engineering may function in unforeseeable ways.

The adhesive plasters used to attach the accelerometers, which had been used previously for children in settings other than hospitals [21,41,42], caused skin reactions in one-third of the children. One of the various reasons for their discomfort may be that they were often lying down under covers in bed for many hours a day, possibly leading to increased sweating and itchy, irritated skin under the adhesive plasters. Perforated adhesive plasters that improve ventilation are a possible solution just as removable Velcro bands could be tested, which would also allow staff to reattach accelerometers more easily.

Accelerometer data showed that the children were inactive for 22 hours a day. Due to the small sample size, their inactivity reflects individual diseases and treatments more than a general issue. The low level of activity means a few extra daily steps would be beneficial and that 1, not 3, accelerometers are likely sufficient to provide a detailed activity profile. The level of inactivity, however, resembles that of hospitalized adults [22] and calls for further investigation.

### Strengths and Limitations

We followed all relevant guidelines when planning and conducting this feasibility study. Some of the strengths include asking the children about their motivation and attitude towards the intervention rather than assuming it would be fun and motivating just because the app is playful. One limitation is that we only surveyed the children, adolescents, and health care professionals about their opinions, experiences, and attitudes concerning the intervention instead of using more in-depth interviews to gain greater insights.

### Perspectives

If the objective is to go further by testing the efficacy of this intervention in a full-scale RCT, we propose adjusting the intervention by (1) recruiting from several hospitals, (2) adding measures to reduce the risk of data loss, (3) further developing the app to support all types of mobile phones and operating systems, or providing devices for the duration of the intervention, (4) encouraging participants to use the free Wi-Fi to ensure a proper internet connection, and (5) using a more comfortable method to attach the accelerometer. In addition, future digital interventions should be simplified to minimize cognitive distress. Another suggestion is to hold informational meetings with health care professionals and recruit nurse ambassadors familiar with the intervention to publicize it. Including end users in intervention development would also be useful. To lighten the nurses’ workload, a student assistant could also be employed to facilitate the intervention. Last, the level of physical inactivity reported in this feasibility study is alarming. Moreover, there is currently no known scientific literature evaluating the level of physical activity among hospitalized children, and no reference material exists for this population. We highly encourage future studies to evaluate the activity level of hospitalized children across various health conditions.

### Conclusions

The digital play intervention Monster Gardener demonstrated potential in terms of fostering motivation for physical activity during pediatric hospitalization; however, the current version was deemed infeasible for implementation in this setting, based on the 8 feasibility focus areas by Bowen et al [18]. Our findings indicated that various organizational, technological, and practical issues must be addressed to adjust and improve the intervention prior to conducting effectiveness testing. Finally, future studies should focus on simple digital play interventions and the dynamic involvement of end users in development.

## Supplementary material

10.2196/58019 Multimedia Appendix 1 Description of the app, Monster Gardener.

## Abbreviations

API: application programming interface
AR: augmented reality
CFIR: Consolidated Framework for Implementation Research
CONSORT: Consolidated Standards of Reporting Trials
IMI: Intrinsic Motivation Inventory
RCT: randomized controlled trial
REDCap: Research Electronic Data Capture
TAM: Technology Acceptance Model
