# Supplementary material for: Investigating Challenges in Implementing a Digital Play Intervention in a Complex Organization Across Pediatric Departments: Non-Randomized Controlled Feasibility Trial
Source: JMIR Rehabil Assist Technol. 2025 Jul 8;12:e58019. doi: 10.2196/58019 (PMC12262151; doi:10.2196/58019)

Multimedia appendix for

Investigating Challenges in Implementing a Digital Play Intervention in a Complex Organisation Across Paediatric Departments: A Feasibility Study

Lærke Winther, Michelle Stahlhut, Derek John Curtis, Christian Have Dall, Thomas Leth Frandsen, Jette Led Sørensen

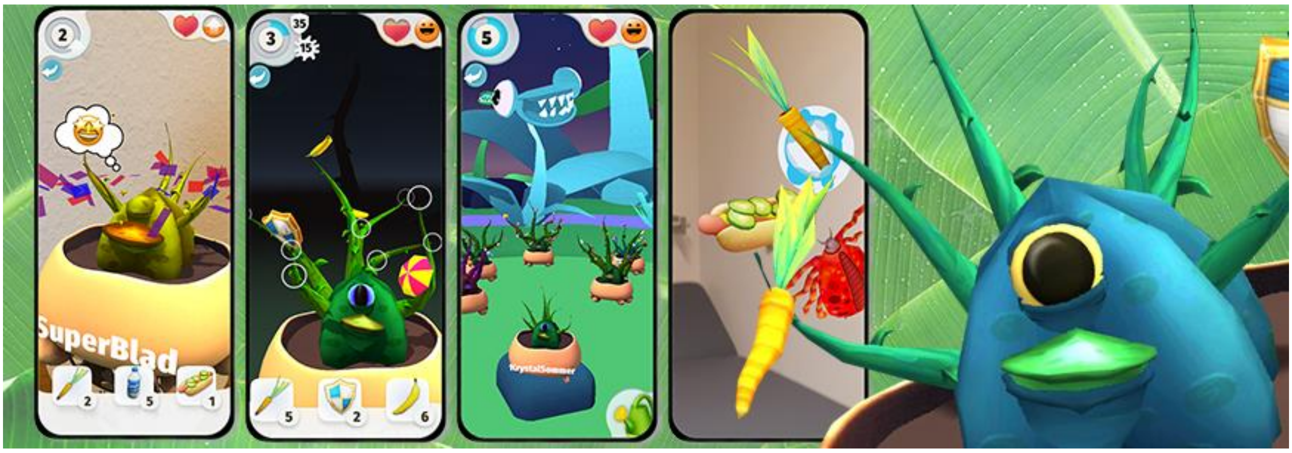

Augmented reality (AR) technology in the app merges the real world with virtual elements, encouraging physical activities like walking and boxing, and the camera lens in users' phones captures the real world combined with digital elements integrated into the surroundings. Designed to be playful and engaging to counter the negative impacts of hospitalisation on children's health, Monster Gardener invites children to act as gardeners for animated monster plants.

The children were provided with a personal AR code that would hold their own personal Monster plant. When they open the app and scan their personal code, the Monster plant emerges.

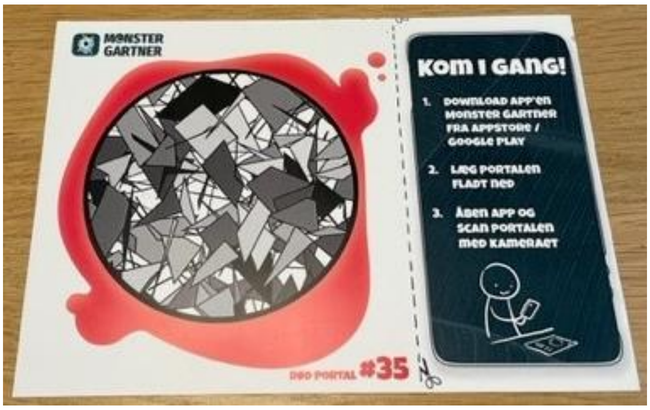

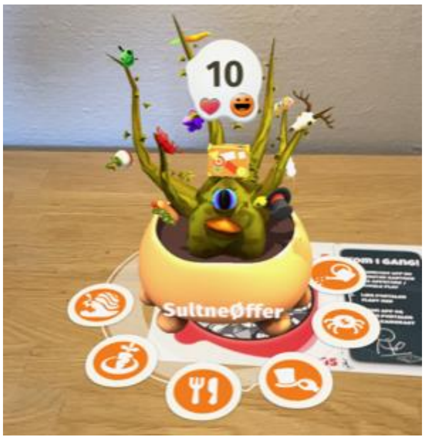

The app comprises six mini-games: three for adaptation (feeding, decorating and social activities) and three five-minute mini-games that encourage physical activity. These games, including two walking activities and an activity for the upper extremities, were disguised and embedded in the digital play to encourage movement in an entertaining digital context.

The three mini-games for physical activity are “Snail war”, “Treasure hunt” and “Food walk”:

In the Snail War mini-game, an A3 poster with an AR code was hung on the wall at the reception in each department. When you scanned the AR code, a shelf would appear on the wall with a large snail trying to reach your plant. To defeat the snail, you had to poke and swipe in different directions for 5 minutes. The physical activity from this game was the getting out of bed and moving to the poster.

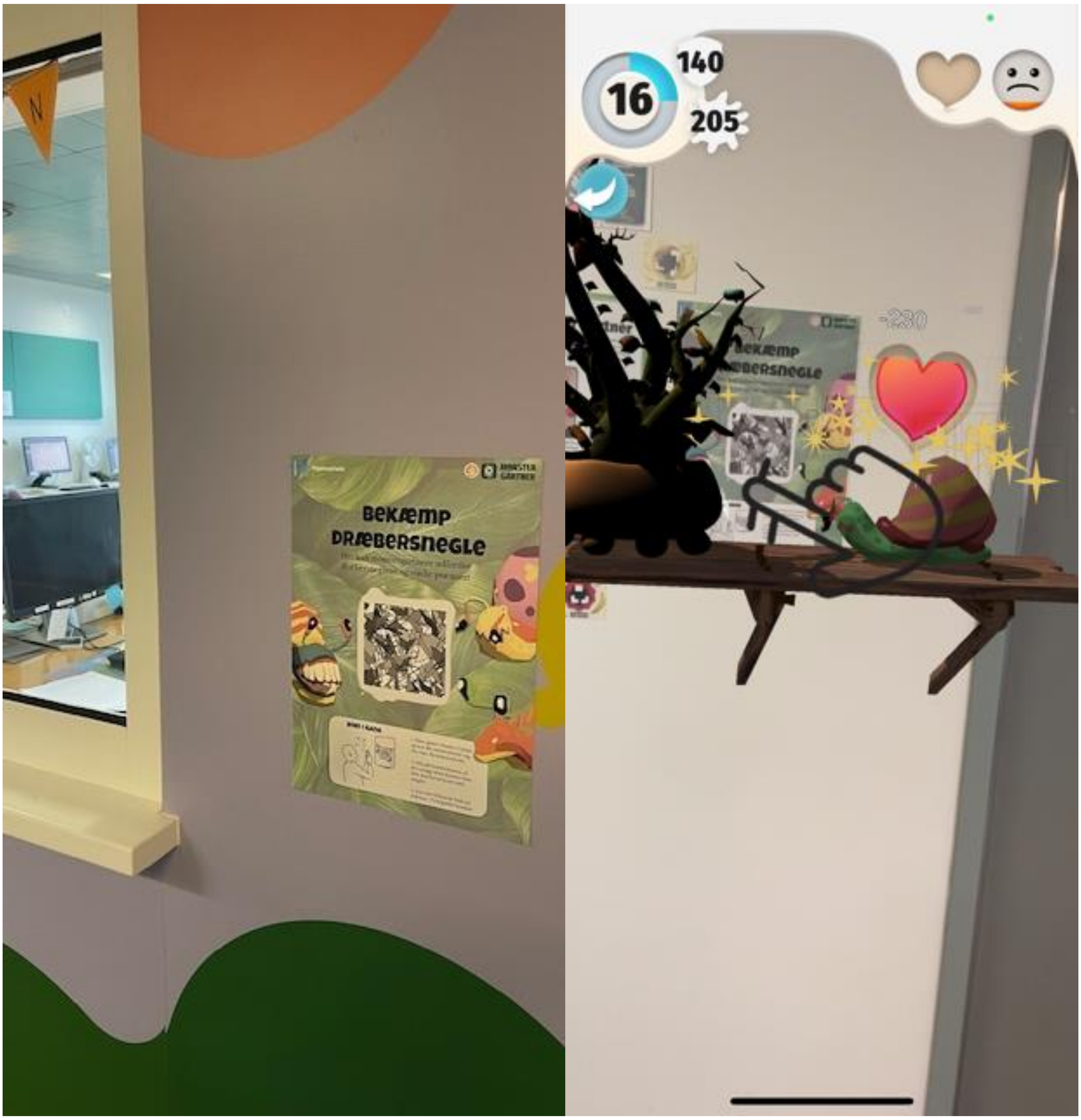

In the Treasure Hunt mini-game, the player was presented with a map with four quests. The story line was, that you must protect your Monster-seeds and bring them to a magic tree at the end of the map to

receive an award. Each quest represented a mini-game where you had to move you arms to interact with the game, i.e. by boxing flies away from your seeds that were hanging in a net.

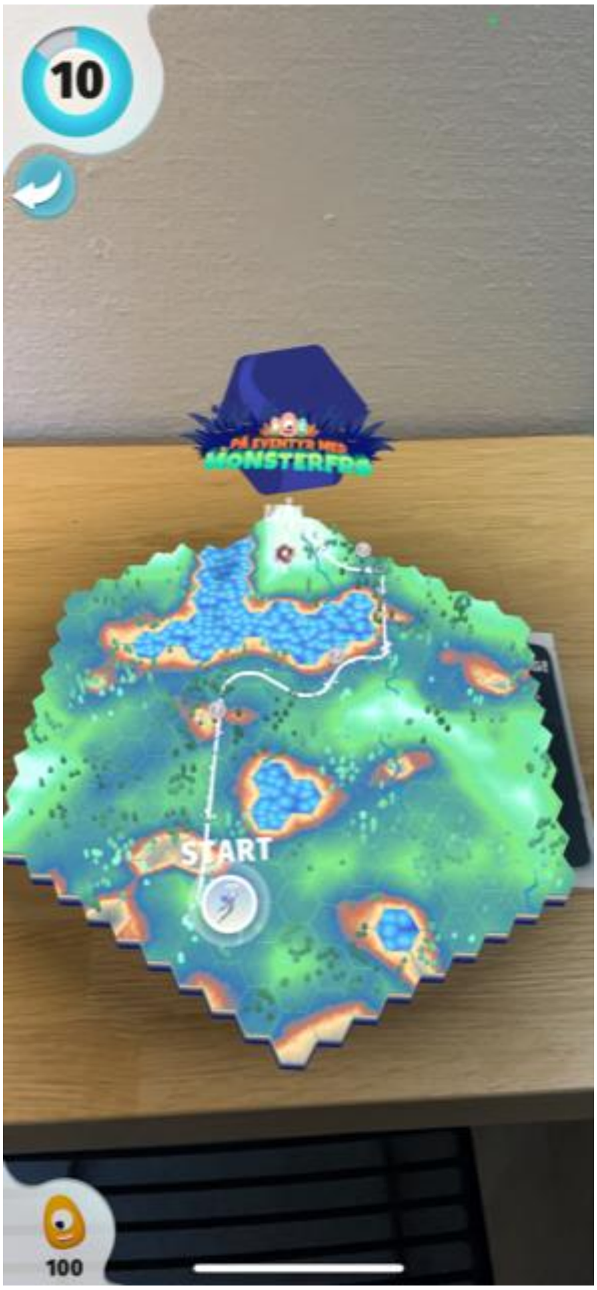

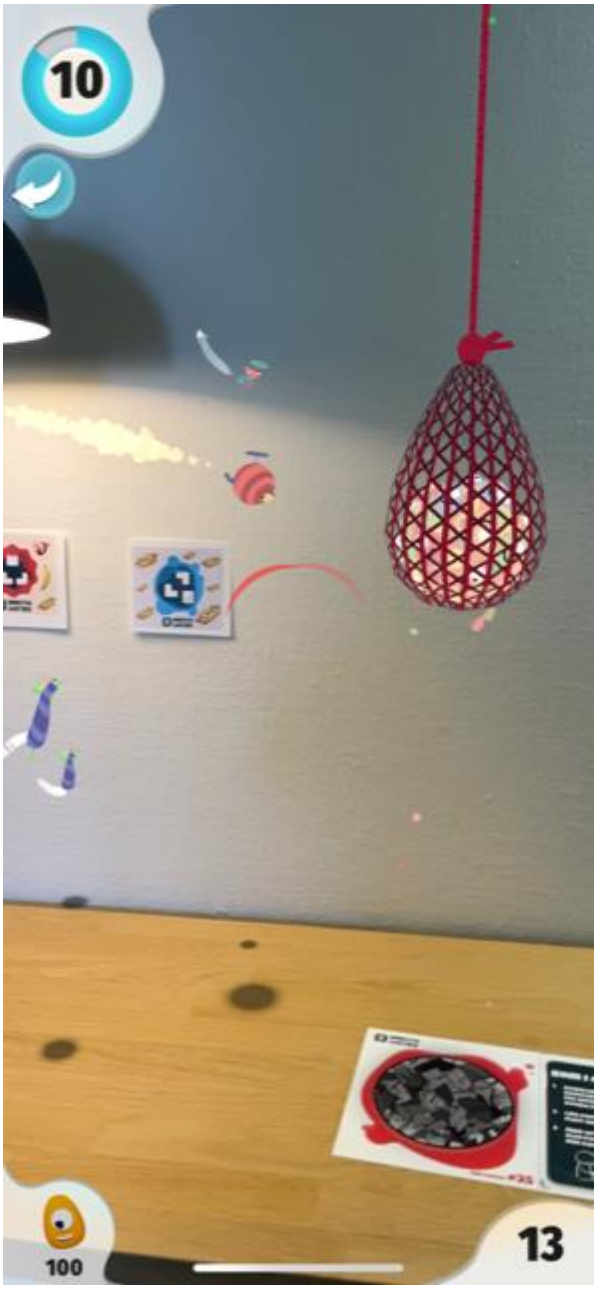

In the Food Walk, the player had to find food for its plant. The food was hidden in AR codes that were strategically placed in the children's hospital rooms, around the hospital wards, and on staff uniforms. Food and decorative items for the monster plants would appear on the children's phones when scanning the AR codes, nudging them to get out of bed to find the codes. The AR codes were color-coded as red, yellow, and blue, positioned at one end, the middle, and the opposite end of the ward, respectively. Each app quest required scanning at least two AR codes, ensuring a minimum walk from the middle to an end of the ward. Upon completing a quest, children received a new quest with an additional AR code to advance further

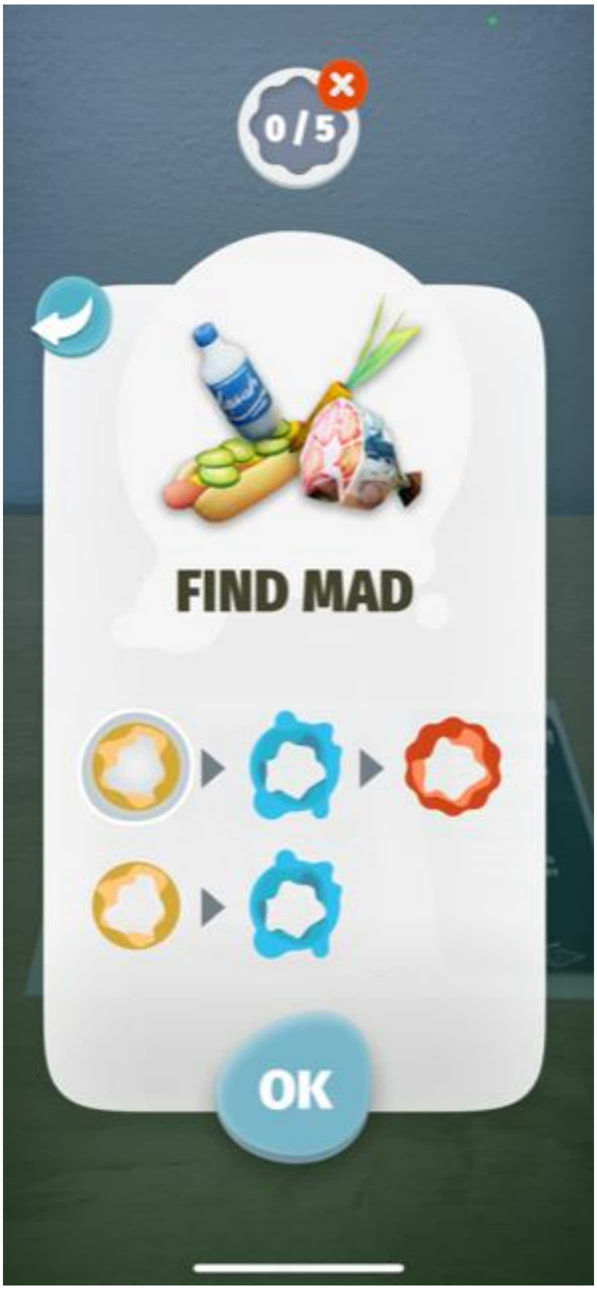
0 / 5
FIND MAD
OK

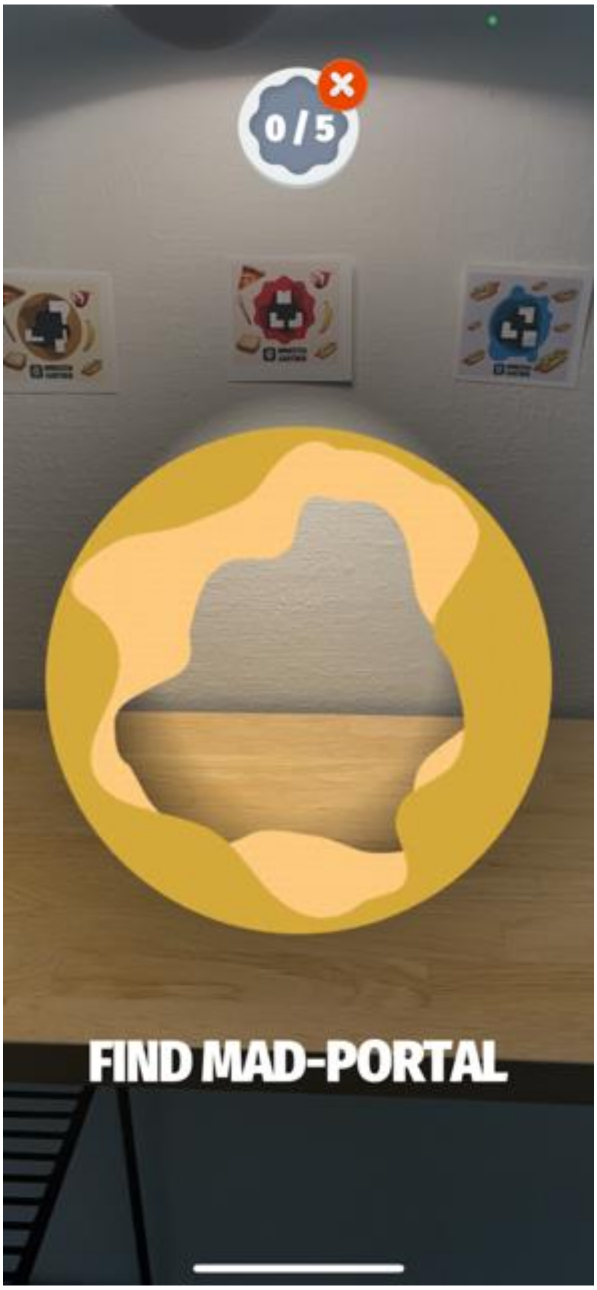
0 / 5
FIND MAD-PORTAL

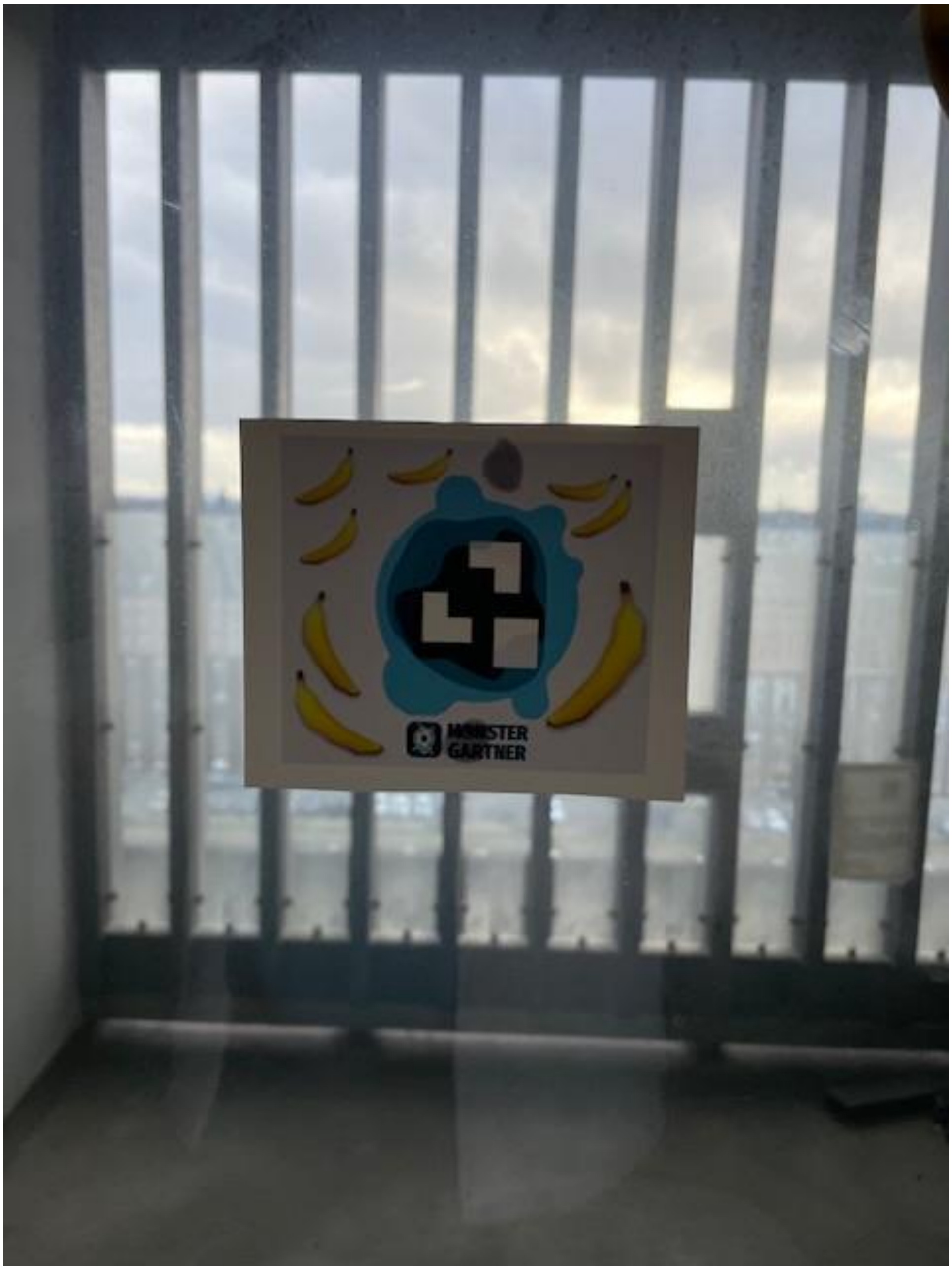

Supplement: Multimedia Appendix 1 [file rehab-v12-e58019-s001.pdf]
